# A small footprint couch‐top support device for image‐guided radiotherapy of heavy patients

**DOI:** 10.1002/acm2.13788

**Published:** 2022-10-20

**Authors:** Huixiao Chen, Kevin Morley, Ronnie Rodriguez, Emily Draeger, Muhammad Shafiq ul Hassan, Zhe (Jay) Chen

**Affiliations:** ^1^ Department of Therapeutic Radiology Yale School of Medicine New Haven Connecticut USA

**Keywords:** couch‐top support, heavy patient, image‐guided radiotherapy, linac

## Abstract

**Purpose:**

Patients with body weights close to or above 400 lbs present unique challenges in radiation therapy since the weight limit of most treatment couches decreases as the couch‐top extends toward the treatment gantry. The purpose of this work was to develop a small footprint couch‐top support platform to safely perform image‐guided radiotherapy (IGRT) for extremely heavy patients.

**Methods:**

One way to protect the couch‐top from damage and prevent a catastrophic breakdown is to provide additional support as the couch extends toward the treatment gantry. To allow a maximal range of gantry movement, a small‐footprint adjustable jack stand, placed underneath the couch‐top, was chosen and modified from a commercial jack stand (with 1100 lbs capacity). The couch could be easily extended longitudinally and laterally with a modified 8‐ball‐transfer plate mounted at the top. The operation of a couch‐top support platform was used for two heavy patients after phantom testing. kV and MV imaging options and ranges were quantified.

**Results:**

The custom‐constructed couch‐top support platform was found to provide stable support with smooth couch shifts. The small footprint allowed gantry rotation from 133° to 227°, which would allow both fixed beam radiotherapy and partial‐arc volumetric modulated arc therapy (VMAT). For IGRT, orthogonal 2D kV‐kV image pairs with source angles of 40^o^ and 130^o^ were acquired and tested successfully. With the support platform, two clinical cases with patient weights greater than 415 lbs were successfully treated with image‐guided partial arc VMAT radiotherapy. The study demonstrated the safety and efficiency of using this new couch‐top support platform to prevent couch failure from treating heavy patients.

**Conclusions:**

A new couch‐top support platform has been designed, assembled, and tested for IGRT. The new support platform is easy to use, cost‐effective, and allows extremely heavy patients to be treated safely and robustly with IGRT and VMAT.

## 1 INTRODUCTION

Obesity has become a serious health issue in the United States, with nearly 35% of Americans being categorized as obese.^1^ For obese cancer patients, the impact of diagnosis and treatment is a recognized and growing challenge.^2^ Over the last two decades, in addition to surgery and chemotherapy for the treatment of cancer, the use of volumetric‐modulated arc radiation therapy (VMAT), an advanced radiation treatment technology, has risen dramatically — with VMAT used for up to 42% of cancers treated with radiation in the United States.^3^ However, several studies of external‐beam radiation therapy have reported the technical issues in delivering radiation therapy to obese patients, such as difficulty with daily setup and an increased likelihood of shifts in tumor location among obese patients.^4^ Particularly, heavy patients with body weights close to or above 400 lbs present unique challenges in radiation therapy, since the weight limit of most linear accelerator (linac) treatment couches is around 181kg (400lb). In addition, the couch weight limit reduces when the couch‐top extends toward the treatment gantry.

The nominal weight limits for the couch‐tops used in our department, for example, are 155 kg (341 lbs), 159 kg (350 lbs), and 200 kg (440 lbs) for the Qfix Calypso KVue couch‐top (Perfectpitch 6 DoF) on Varian TrueBeam, the Exac Trac couch‐top on Varian Trilogy, and the iBEAM Evo couch‐top on Elekta Synergy, respectively.^5^, ^6^, ^7^ For the Elekta iBEAM Evo couch‐top, although the maximum load distributed evenly over the top is 200 kg (440 lbs), the maximum load at the cranial end of this couch top (with no extension attached) is only 100 kg (220 lbs). Note that the maximum load is the total load, including the weight of the patient, any attachments, and the weight of accessories. Treatment setup devices and accessories can weigh up to 15 kg (33 lbs),^7^ which can further reduce the maximum weight of the patient that the treatment couch can support.

While there are technical challenges and growing clinical needs to treat heavy patients, only one limited study was published by Patrick Towns et al. in 2004.^8^ In that study, Towns et al. reported the safety issue for radiotherapy treatment of heavy patients, particularly if the couch has been in use for several years and has seen significant wear and tear. They explained the mechanical causes of couch failure and demonstrated the design and implementation of a lifting apparatus (Genie Lift, Genie Industries, Redmond, WA).^9^ to help support the treatment couch for heavy patients. The support lift described by Towns et al. provided an invaluable contribution to the treatment of obese patients at the beginning of the 20th century when onboard imaging systems were not mainstream. During that period, beams with fixed gantry and without image guidance were used in the routine clinical practice of radiotherapy. However, as technologies in image‐guided volumetric modulated arc radiotherapy were introduced and advanced over the past decade, the lifting apparatus reported by Towns et al., with the dimensions of about 50 × 50 × 200 cm, limits the implementation of on‐board imaging systems and the rotational beam delivery for modern radiotherapy.

To meet the growing clinical need for additional couch support and solve the challenge of radiotherapy for heavy and obese patients, we have developed a small footprint couch‐top support platform to safely perform image‐guided radiotherapy (IGRT) for extremely heavy patients. The design and development of this new device, as well as the initial clinical experience of using this device for two heavy patients, are reported.

## 2 MATERIALS AND METHODS

### 2.1 A new couch support device

**FIGURE 1:**
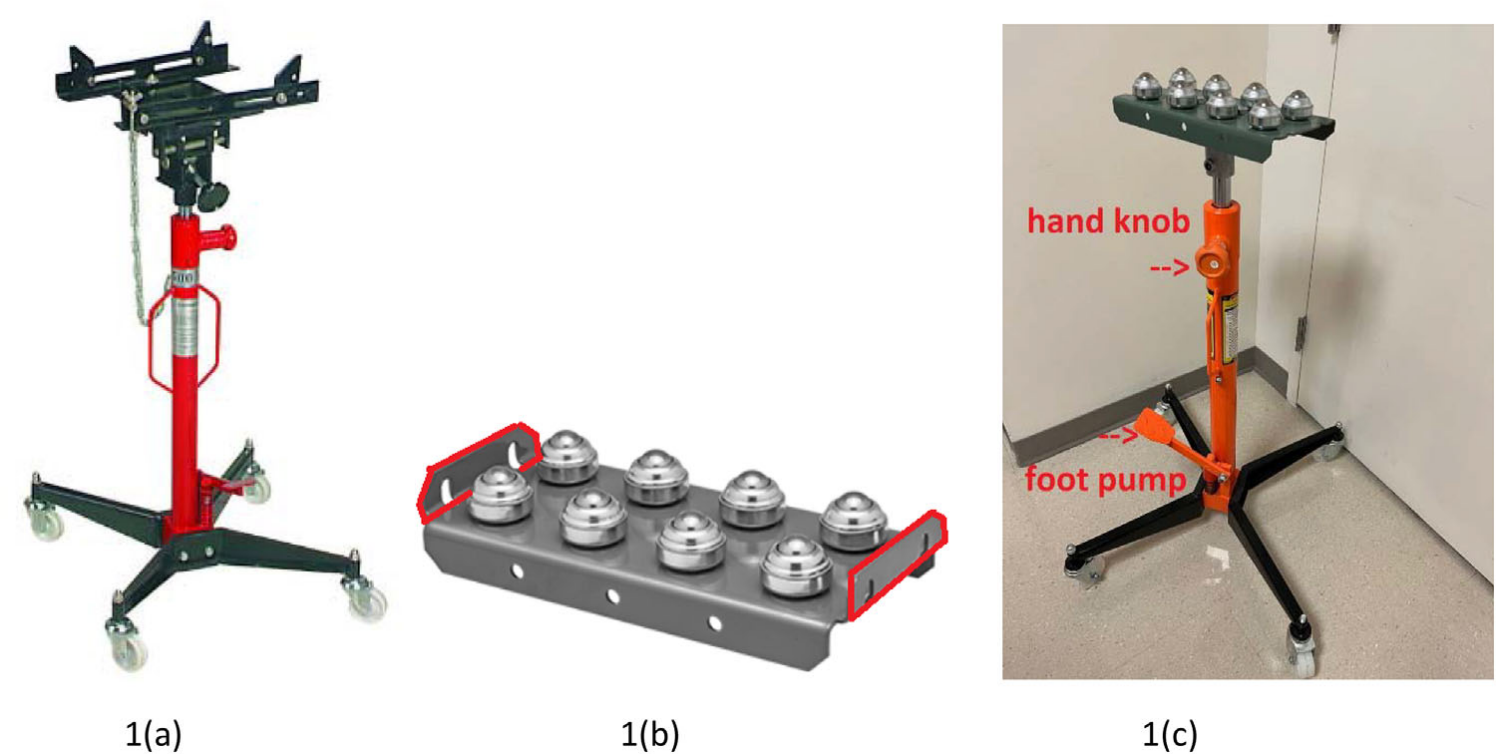
(a) Commercial 1100 lbs high lift transmission Jack; (b) 8‐ball transfer roller (side metal pieces outlined in red removed); (c) Modified patient support device

The in‐house developed couch support device (Figure 1c) consisted of two main parts: the main support transmission jack frame (Figure 1a) and an add‐on top roller plate (Figure 1b). A high lift transmission jack with 1100lb capacity (Habor Freight Company, Calabasas, CA) was modified by removing the original upper black saddle. After the removal of the upper metal portion, the transmission jack was 127.0 cm in height without the inner support pole extended. An 8‐ball transfer roller plate (McMaster‐CARR Company, Elmhurst, IL or Douglasville, GA) was mounted at the top of the transmission jack, with the side metal pieces (outlined in red in Figure 1b) removed to allow multidirectional movement. The transfer roller plate was 15.3 cm wide and 33.0 cm long and included 8 powder‐coated steel balls (7.6 cm spacing) with a Zinc plated steel housing and a weight capacity of 520 lbs. A metal sleeve with a 5.1 cm inner diameter was welded to the center of the backside of the roller plate for a seamless connection to the bottom support pole of the transmission jack. The whole platform (Figure 1c) allows for fast, smooth, and durable translation in the x (lateral) and y (longitudinal) directions of the supported couch top. In addition, the inner steel pole of the support jack was marked with scales of different heights for the readings of relative couch vertical adjustment. Also, the convenient foot‐operated pump (labeled in Figure 1c) on the device enables users to keep their hands free while extending the jack height, while the spring‐loaded release valve automatically halts lowering when pumped. The safety hydraulic valve lowers only when the user's hand is on the knob (labeled in Figure 1c). Furthermore, this device has an extra stable wide base with four swivel casters for easy positioning. There are no brake locks needed for the four‐foot rollers as the device's weight and mechanical design provide sufficient resistance to ensure its stability. Overall, the device is small and portable, with the main limitation being the space occupied by the load‐bearing column. As this column has a diameter of only 5.1 cm, it allows almost three‐quarters of a full range of gantry movement (from 133° to 227°).

### 2.2 Clinical treatment summary of two heavy patients

The first case was a 37‐year‐old female patient, with a body weight of 188 kg (415 lbs), diagnosed with right chest wall recurrence of classical Hodgkin's Lymphoma, stage IV. After a series of chemotherapy and bone marrow transplant, the patient was referred for consolidative radiotherapy. The physician prescribed image‐guided VMAT treatment with daily fractional doses of 2 Gy and a total dose of 36 Gy (18 fractions). The second case was a 61‐year‐old male, with a body weight of 187 kg (412 lbs), who was diagnosed with prostate cancer. The physician prescribed image‐guided VMAT treatment through the fiducial match, with daily fractional doses of 2.5 Gy and a total dose of 70 Gy (28 fractions).

### 2.3 Treatment machine and couch weight limits

An Elekta Synergy Linac (Elekta Limited, Crawley, UK) equipped with an iBEAM EVO couch‐top was selected for treatment. Of the linacs available at our institution, this machine offered the highest couch weight capacity, with a maximum load (distributed evenly on the couch‐top) of 200 kg (440 lbs). However, the maximum load at the cranial end of this couch‐top (with no extension attached) is only 100 kg (220 lbs). For the patient with chest wall treatment, the couch needed to be longitudinally shifted further toward the gantry, resulting in even less support from the original couch column.

### 2.4 Image‐guided radiotherapy

**FIGURE 2:**
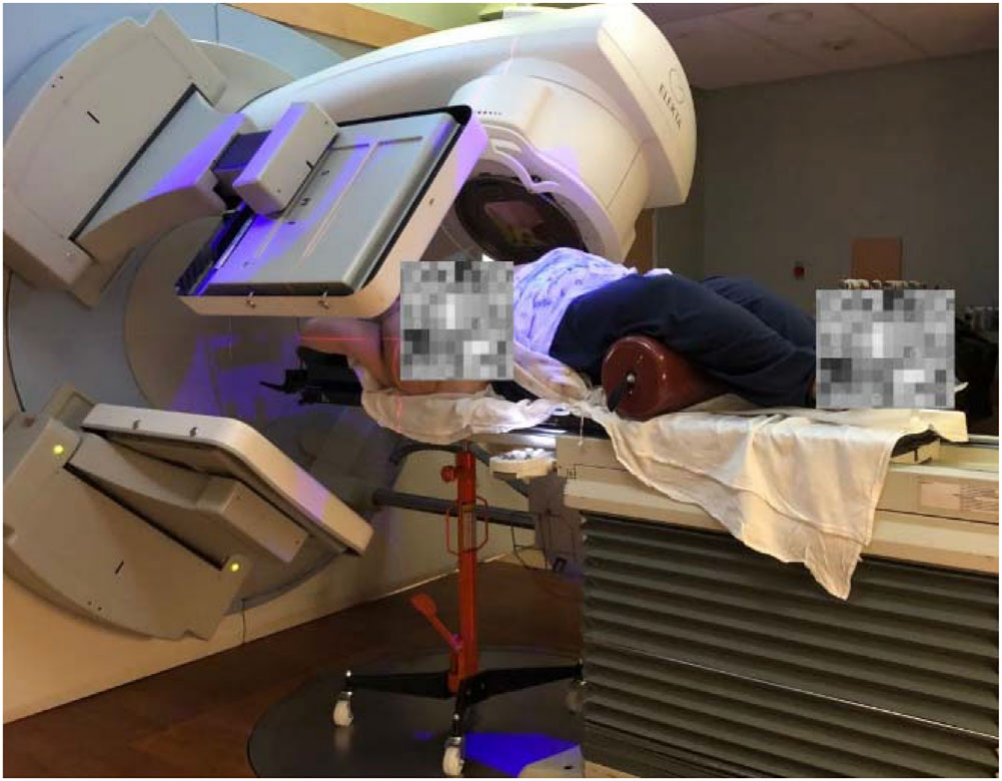
kV image acquisition before beam delivery

**FIGURE 3:**
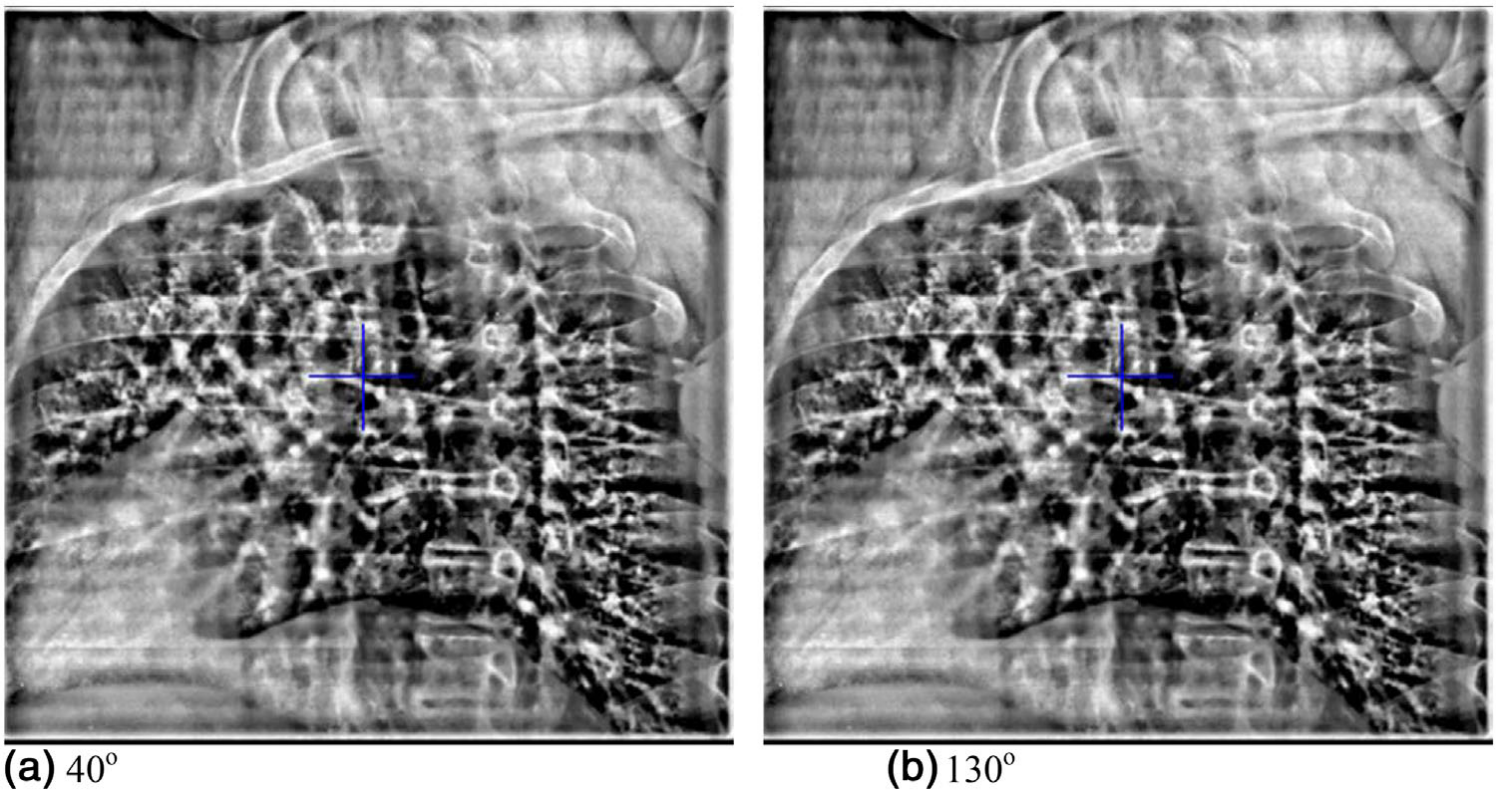
kV image pair acquired for the first patient at 40^o^ and 130^o^, respectively

IGRT for the first patient involved 1) orthogonal kV imaging and 2) Surface image aided initial setup and deep inspiration breath hold (DIBH) gated beam delivery. The patient was initially set up with traditional 3 marked points and CRAD (C‐RAD AB, Sweden) cPosition alignment. Afterward, orthogonal imaging was carried out using the kV imaging system on the Elekta Synergy Linac, with a pair of kV images acquired at source angles of 40^o^ and 130^o^ as the posterior direction was blocked by the support device (Figure 2). A kV image protocol of 140 kV and 80 mAs was selected due to the patient's size. The kV images acquired for position verification are shown in Figure 3.

**FIGURE 4:**
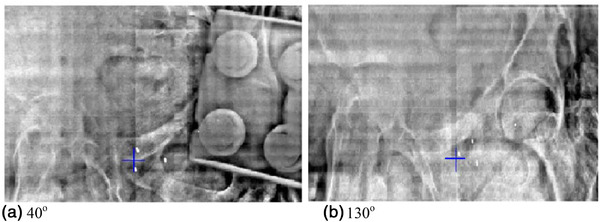
kV image pair acquired for the second patient at 40^o^ and 130^o^, respectively

For the second case, kV IGRT was accomplished through the registration of three fiducials on the live 2D images to the digital reconstructed radiographs. Figure 4 shows the kV images acquired for the second patient's position verification.

The information pertaining to the patients and their treatment modalities is summarized in Table 1.

**TABLE 1:** Summary of patient information and their treatment modalities

	Age/gender	Body weight	Treatment site	Prescribed dose	Treatment technique/IGRT
Patient 1	37/F	188 kg (415 lb)	Right chest wall	36 Gy (2.0 Gy × 18)	Gated VMAT Orthogonal kV images (40^o^ and 130^o^) + surface imaging with DIBH
Patient 2	61/M	187 kg (412 lb)	Prostate	70 Gy (2.5 Gy × 28)	VMAT Orthogonal kV images (40^o^ and 130^o^) with three fiducials

### 2.5 Treatment planning and beam delivery

The two treatment cases utilized VMAT planning with partial arcs, with the first plan consisting of two anterior half arcs between 90^o^ and 270^o^ (clockwise and counter clockwise, respectively), and the second consisting of three partial arcs between 240^o^ and 120^o^ (two clockwise and one counter‐clockwise). Partial arcs were chosen to not only meet the prescribed dose distribution objectives and organ at risk constraints but also to avoid the posterior jack blocked area. To minimize lung and heart dose for the first patient, DIBH gated beam delivery was also implemented with the CRAD, with the reference image acquired during CT simulation.

The detailed patient alignment for the first case and beam delivery workflow with the support device is summarized below, with the second case following similar steps as the first minus CRAD image acquisition (steps 4 and 5):

1. Align couch support device with the predefined floor marks
2. Align the patient on the couch and raise the couch according to the three‐point skin markers
3. Adjust couch support jack height to fit the couch‐top
4. Check the initial setup by the CRAD cPostion feature
5. Adjust the couch position in the treatment room if needed
6. Acquire orthogonal kV images at 40° and 130°
7. Adjust couch position in the treatment room if needed
8. Retract the kV source arm and imager panels (including kV and MV)
9. Deliver arc beams

### 2.6 Quality assurance (QA) for image‐guided VMAT using the couch‐top support device

Before the first day of treatment, a dry run test was performed to validate the collision clearance for the orthogonal kV imaging and treatment fields. The positions of the four swivel casters were marked on the floor for accurate daily device positioning for subsequent fractions.

To verify the mechanical isocenter's offset couch walk‐out was checked daily by rotating the couch through 180^o^, and marking the end of the front pointer every 45^o^ on millimeter scale graph paper at 100 cm SSD. The diameter of the enclosed circle was recorded. Additional QAs were completed in the middle of the patient's treatment course and after the patient's last treatment including couch shift accuracy and Winston‐lutz test. The precision of absolute couch readings and shifts was verified using the comparison with the predefined absolute readings and shifts by a calibrated ruler. The coincidence of the couch mechanical isocenter with the radiation isocenter was verified by performing a Winston‐Lutz test^9^. We took 2cm x 2cm MV images through the electronic portal imaging device with the Elekta 8 mm ball‐bearing phantom mounted to the treatment couch^10^. The offsets in the vertical direction and the horizontal direction between the couch and radiation isocenter were recorded.

The quality assurance procedure, in addition to our routine linac QA, for image‐guided VMAT using couch‐top support for heavy patients is summarized in Table 2.

**TABLE 2:** QA for image‐guided volumetric modulated arc therapy (VMAT) using a couch top support for heavy patients

Before treatment	After treatment
Dry run test for the collision clearance Mark four swivel casters on the floor	Couch translation indicator in longitudinal, lateral, and vertical directions (<1mm). at first three fractions, middle, and after the treatment course Couch walkout check using projection of cross hair (<1mm), at first three fractions, middle, and after the treatment course Wintz‐Lutz test (<1mm), in the middle and after the treatment course

## 3 RESULTS

### 3.1 The new couch‐top support platform

A new small‐footprint couch‐top support platform was successfully built which possesses the following features:

1. Weight capacity: 520 lbs for roller transfer plate (1100 lbs for the adjustable support column), which is capable of supporting the heaviest patient in our department thus far;
2. Size: Small footprint with less than 6 cm in diameter for the main support column, which allows not only fixed gantry but also arc rotational beam delivery;
3. Mobility: The couch‐top support platform is easy to move in or out of the treatment vault and to position with four swivel casters attached to the wide base;
4. Couch‐top support and maneuverability: The height of the support platform can be adjusted easily with a foot paddle to provide constant support to the couch top; the roller transfer plate atop the support platform not only provides robust support for the couch‐top but also allows the couch‐top to be translated easily in both the longitudinal and lateral directions; it enables image guidance and subsequent couch shifts with high precision;
5. Cost: Less than $400 for the commercial lift transmission jack and the roller transfer plate;
6. Durability: The device appears to be very robust after initial testing and use on two patients.

### 3.2 Image‐guided VMAT treatments

The small footprint couch‐top support platform was used successfully in completing 18 and 27 fractions of VMAT treatments for two obese patients with precise image guidance, respectively. The couch‐top support device allowed the gantry to rotate from 133^o^ to 227^o^, and can be used for either fixed beam treatment or partial‐arc VMAT. For the clinical cases, half arcs between 90^o^ and 270^o^ were found to be sufficient. Orthogonal 2D kV‐kV images with source angles of 40^o^ and 130^o^ were acquired successfully for image‐guided positioning. Lateral MV images were also successfully acquired, though MV imaging was not prescribed for these two cases. Figure 2 illustrates the kV planar images acquired in the orthogonal directions, while Table 3 shows the detailed daily couch shifts after kV imaging for the first and second cases, separately. The time spent for the first patient's daily treatment session including alignment, couch shifts, and DIBH gated beam delivery was shortened from 45 min on the first day to 25‐min sessions from the 3rd fraction and on. For the second patient, only 15 min was needed for the daily alignment and beam delivery. Additionally, the offsets from the Winston‐Lutz testing in the vertical direction and the horizontal direction between couch isocenter and radiation isocenter were all within 1 mm after heavy patient treatments. The differences in Winston‐Lutz tests before and after heavy patient treatment were all within ±0.2 mm. The couch shift precision and mechanical isocenter verification showed consistency regarding the absolute and relative shift readings, as well as the maximum displacement to the radiation isocenter.

**TABLE 3:** Couch support device aided daily couch shifts for two patients

			**Min**	**Max**	**Average**
	Patient 1	S/I (+/−)	−1.5	1.3	−0.4 ± 0.8
		L/R (+/−)	−0.4	1.8	0.4 ± 0.6
Shifts (cm)		A/P (+/−)	−0.6	1.7	0.4 ± 0.6
	Patient 2	S/I (+/−)	0.3	−1.3	−0.6 ± 0.3
		L/R (+/−)	1.5	−0.7	0.2 ± 0.7
		A/P (+/−)	0.6	−0.7	0.0 ± 0.3

This newly constructed couch‐top support platform was found to be very easy to use, reliable, and provided stable support with smooth and fast couch shifts.

## 4 DISCUSSION

Many reports and recent clinical consultations have shown an increase in the number of obese cancer patients, as well as the challenges for radiotherapy of heavy patients. The upper weight limit of treatment couch tops is one of the hurdles preventing advanced treatment planning for these patients. Previous couch support devices and techniques such as the giant genial lift on the side of the couch, or large rigid boxes underneath the couch, are no longer compatible with the rapid development of IGRT. Therefore, providing a new, portable, small footprint couch support device to allow safe treatment of heavy patients undergoing IGRT has become a critical clinical priority. To develop a couch support device allowing for the maximum range of gantry rotation and smooth couch shifts in the X, Y, and Z directions, literature, and commercial high lift transmission jacks were extensively reviewed. To our knowledge, the work presented here represents the first couch support device compatible with image guidance and offers excellent flexibility for performing couch shifts. Moreover, with manageable clinical treatment times (average 25–30 min) after dry run testing and the first few treatment fractions performed by the treatment team members, including a medical physicist and therapists, the clinical utility of this support device is very promising.

There were several questions we noticed during the clinical implementation of these devices. First, for the image guidance of one patient, it appeared that the roller plate was included partially in the image acquisition, although it did not affect the image registration accuracy since the image guidance was based on the fiducial markers. The appearance of the roller plate in image acquisition can be minimized by adjusting the location of the couch support device and the orientation of the roller plate on the treatment simulation day. For example, while we typically position the roller plate with its long axis along the transverse direction of the couch‐top, it can also be positioned with the long axis along the longitudinal direction (without loss of mechanical support integrity) to minimize the appearance of the roller plate in the acquired images. In addition, the location of the support device (hence the roller plate) can be optimized on the simulation day to keep the roller plate out of the imaging field of view or with minimal impact on acquired images. With these maneuvers and with the use of a large imaging field of view, we anticipate the acquired orthogonal images would be able to provide sufficient information for an accurate 2D image registration for most if not all patients. The optimal position of the support device and roller plate determined on the treatment simulation day can be reproduced during the treatment course by using the markers on the floor for the foot of the device and the tapes underneath the couch for the position of the roller plate. Second, the average daily shifts of these two heavy patients’ alignments were less than 0.5 cm with a maximum daily shift of 1.8 cm. They are very reasonable and acceptable even compared to our SBRT cases. While this is a good indication that the patients’ setup was robust and reproducible, it did not answer the question of whether larger shifts could present any issues when using this support device. To address this, we performed tests for large shifts (e.g., 5 cm) with a heavy phantom (450 lbs) on the couch, there was no issue noted for smooth couch shifts. The support device can also be moved accordingly with the couch shift to maintain support stability if larger shifts are needed. For all these test scenarios, we did not observe any issues. Another concern is regarding the roller plate being included in the beam's path for some beams (e.g., AP beam) and how much this could affect the dose distribution. As the irradiations are all from the anterior and side directions, the roller plate (if in the beam path) would always be on the exit side of the beam. The dosimetric effect from the roller plate would mainly be due to backscattered radiation. We performed phantom tests with ion chamber point measurements in the center of the field and at different depths (1, 5, 10, 15, and 20 cm) from the couch top, with and without the support device fully present in the irradiation field (10 × 10 cm, the gantry is 0^o^, beam energies are 6, 10, and 15 MV respectively). The average dosimetric deviation is less than 0.4%, in both cases with and without a support device present, and with a maximum deviation of 0.8%. This could be considered a negligible effect in the clinic.

Another debate is whether heavy patients, slightly beyond the manufacturer's upper weight limit, can be treated without a support device. Over the last few years in our department, we have had an increased number of consultations regarding the feasibility of radiotherapy for heavy patients, such as 460 and 500 lbs. In our clinic, a 430 lbs head and neck patient was treated in 2019 without a support device as the couch did not need to extend in the superior direction. Note that this patient's weight was within our Elekta couch's weight tolerance. However, after the total treatment course of 30 fractions, the couch's mechanical isocenter walked out by 3 mm, and maintenance was required immediately. This demonstrated that, for a couch that has been in service for a number of years and has seen significant wear and tear on its mechanical parts, the user has to be more diligent, even if the weight is barely within tolerance. In addition, vigilance is also needed during each treatment session. Couch shifts must be performed in the treatment room with the implementation of a support device, and imaging panels must be folded before beam delivery.

In this work, we used an orthogonal kV pair for image registration instead of using a classical AP – Lateral image pair due primarily to the presence of the support device underneath the treatment couch. Nonetheless, this configuration shortens the path of X‐rays going through a heavier patient's body. Image quality, therefore, is improved compared to the lateral planar image. However, the clinical limitation of imaging still exists as partial CBCT with more than half an arc is not feasible with this support device on the Elekta machine due to the blocked posterior angles, the extended kV source and kV panel, and the bulky gantry head. These physical obstacles limit the maximum rotational range of the partial CBCT to one‐quarter of the full arc, which causes the truncation of the reconstructed volume. Therefore, we used a 2D planar kV image pair instead.

Another related discussion is whether couch sagging could be improved by the developed support couch top. Though our Elekta couch sagging is within the tolerance of 2 mm from the baseline, it is definitely improved by this support device. It can be improved to 0 mm by verification through the measurement of the couch top relative to the laser projections.

Due to the existence of variation in the designs of the couch‐tops and the onboard image systems, our next step is to investigate the use of this support device for the treatment of obese patients on Varian linear accelerators to explore its wider applications.

## 5 CONCLUSION

In this work, we presented the design, assembly, and clinical implementation of a portable couch support platform for the treatment of obese patients in radiation oncology. This study demonstrated that this new support platform is easy to use, cost‐effective, and allows heavy patients to be safely and efficiently treated with smooth and accurate couch positioning using IGRT.

## AUTHOR CONTRIBUTIONS

Zhe (Jay) Chen contributed to the concept and design of the study. Kevin Morley and Ronnie Rodriguez contributed to the device's assembly and improvement. Huixiao Chen contributed to the clinical implementation and manuscript drafting. Huixiao Chen, Emily Draeger, and Muhammad Shafiq ul Hassan contributed to the data collection and analysis. All authors contributed to the discussions and reviewing of the clinical workflow and manuscript. All authors have approved the final version.

## CONFLICT OF INTEREST

The authors declare that they have no conflict of interest.
